# High-Resolution Mapping of RNA–RNA Interactions Across the HIV-1 Genome With HicapR

**DOI:** 10.21769/BioProtoc.5575

**Published:** 2026-06-20

**Authors:** Haobo Wang, Yan Zhang, Jingwan Han, Dejian Xie, Wenlong Shen, Ping Li, Jian You Lau, Jingyun Li, Lin Li, Grzegorz Kudla, Zhihu Zhao

**Affiliations:** 1 Laboratory of Advanced Biotechnology, Beijing Institute of Biotechnology, Beijing, China; 2 Institute of Microbiology and Epidemiology, Beijing, China; 3 MRC Human Genetics Unit, University of Edinburgh, Edinburgh, United Kingdom

**Keywords:** RNA structure, RNA–RNA interaction, Proximity ligation, HiCapR, Viral genome, HIV-1

## Abstract

The genomes of RNA viruses can fold into dynamic structures that regulate their own infection and immune evasion processes. Proximity ligation methods (e.g., SPLASH) enable genome-wide interaction mapping but lack specificity when dealing with low-abundance targets in complex samples. Here, we describe HiCapR, a protocol integrating in vivo psoralen crosslinking, RNA fragmentation, proximity ligation, and hybridization capture to specifically enrich viral RNA–RNA interactions. Captured libraries are sequenced, and chimeric reads are analyzed via a customized computational pipeline to generate constrained secondary structures. HiCapR generates high-resolution RNA interaction maps for viral genomes. We applied it to resolve the in vivo structure of the complete HIV-1 RNA genome, identifying functional domains, homodimers, and long-range interactions. The protocol's robustness has been previously validated on the SARS-CoV-2 genome. HiCapR combines proximity ligation with targeted enrichment, providing an efficient and specific tool for studying RNA architecture in viruses, with broad applications in virology and antiviral development.

Key features

• This protocol describes a method for capturing and analyzing RNA–RNA interacting fragments from a specific source (e.g., viral RNA).

• This protocol efficiently captures low-abundance RNA from complex samples.

• This protocol provides a new, accurate, and highly sensitive platform for detecting and analyzing specific RNA–RNA interactions, such as those in viral RNAs.

## Graphical overview


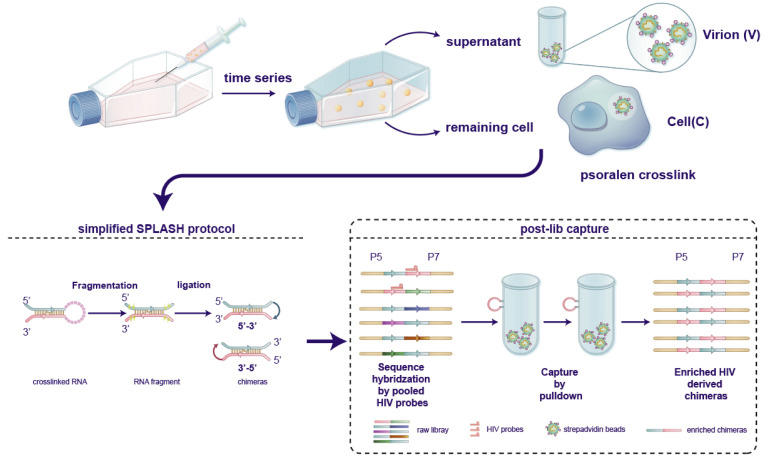


**Overview and procedure scheme.** At the appropriate time point, collect both the supernatant containing live virus and infected cells for psoralen crosslinking treatment. Subsequently, process the crosslinked RNA using a simplified SPLASH protocol. After RNA fragmentation and ligation reactions, obtain chimeric fragments for library construction. Following library capture and post-processing, hybridize the RNA library with specific probes designed for the target virus (HIV-1 in this experiment), to capture and enrich the chimeric fragments. Reproduced from Zhang et al. [1], Mapping HIV-1 RNA Structure, Homodimers, Long-Range Interactions and persistent domains by HiCapR, originally published in *eLife*, https://doi.org/10.7554/eLife.102550.3. This figure is made available under a Creative Commons Attribution 4.0 International license (CC BY 4.0).

## Background

RNA viruses are significant human pathogens that cause a variety of diseases. Their genomes fold into dynamic higher-order structures, enabling precise regulation of critical life processes, such as infection, replication, and immune evasion, all within a limited coding capacity.

High-throughput sequencing technologies for RNA structure detection and sequencing primarily fall into two categories: chemical probe methods and proximity ligation methods [2]. Among these, proximity ligation methods identify interacting RNA regions by capturing and sequencing base-paired fragments, revealing interactions both within and between identical RNA molecules. Experimental methods based on proximity ligation can be broadly classified into two types: those that directly capture RNA–RNA interactions via base pairing, such as PARIS [3], SPLASH [4], LIGR-seq [5], and COMRADES [6]—all of which utilize psoralen-mediated crosslinking—and those that identify protein-assisted RNA–RNA interactions and associated RNA binding proteins (RBPs) through RNA-protein crosslinking, such as CLASH [7], hiCLIP [8], and RIC-seq [9]. The proximity ligation approach has been demonstrated to overcome the RNA length limitations of traditional techniques, proving particularly useful for studying long-range RNA interactions. It has been successfully applied to various viruses, including SARS-CoV-2 [10], Zika virus (ZIKV) [11], Dengue virus (DENV) [12], and Influenza A virus (IAV) [13].

Here, we present the HiCapR (high-throughput capture of RNA interactions) protocol, which builds upon simplified SPLASH methods by incorporating a targeted enrichment step. Like other methods utilizing the reversible, UV-sensitive nucleic acid crosslinker, HiCapR primarily detects the direct RNA–RNA interactions formed through base pairing. The key innovation of HiCapR lies in its fragment enrichment strategy. While methods like PARIS rely on two-dimensional electrophoresis to purify all crosslinked fragments, and others like SPLASH or COMRADES utilize biotin-streptavidin purification either before or after fragmentation, HiCapR implements a post-library capture approach. This means that the targeted enrichment of viral RNA fragments occurs after the cDNA library is constructed. This strategy offers a significant advantage: the amplification during library construction enables highly efficient capture of low-abundance targets, making HiCapR exceptionally suited for studying trace amounts of viral RNA in complex samples. This integrated approach, encompassing RNA extraction, fragmentation, proximity ligation, library construction, and targeted capture, enables a robust study of genomic architecture across various RNA viruses. This protocol has been employed to investigate the structure and dynamics of the complete HIV RNA genome [1].

## Materials and reagents

**Biological materials**

1. Cells and virus (e.g. MT-4 and HIV-1)

**Reagents**

1. PBS, pH 7.4, 1× (Thermo Fisher Scientific, Gibco, catalog number: 10010023)

2. DMSO (Solarbio, catalog number: D8371)

3. EZ-Link psoralen-PEG3-biotin (Thermo Fisher Scientific, Thermo Scientific, catalog number: 29986)

4. 5% digitonin (Thermo Fisher Scientific, Invitrogen, catalog number: BN2006)

5. RNeasy Midi kit (Qiagen, catalog number: 74104)

6. UltraPure DNase/RNase-free distilled water (Thermo Fisher Scientific, Invitrogen, catalog number: 10977015)

7. Chemiluminescent nucleic acid detection module (Thermo Fisher Scientific, Thermo Scientific, catalog number: 89880)

8. ShortCut RNase III (New England Biolabs, catalog number: M0245S)

9. MagicPure RNA beads (TransGen, catalog number: EC501-01)

10. RiboLock RNase inhibitor (40 U/μL) (Thermo Fisher Scientific, Thermo Scientific, catalog number: EO0381)

11. SUPERase·In RNase inhibitor (20 U/μL) (Thermo Fisher Scientific, Invitrogen, catalog number: AM2694)

12. T4 RNA Ligase 1 (ssRNA ligase) (New England Biolabs, catalog number: M0204S)

13. T4 polynucleotide kinase (T4 PNK) (New England Biolabs, catalog number: M0201S)

14. miRNeasy Micro kit (Qiagen, catalog number: 217084)

15. SMARTer Stranded Total RNA-Seq kit v2 (Takara Bio, Clonetech, catalog number: 634411)

16. TargetSeq One Hyb & Wash kit (for Illumina) (iGeneTech, catalog number: C10602)

17. TargetSeq HIV panel (iGeneTech, catalog number: PH2000552)

18. High-sensitivity RNA ScreenTape (Agilent, catalog number: 5067-5579)

19. High-sensitivity RNA ScreenTape sample buffer (Agilent, catalog number: 5067-5580)

20. High-sensitivity RNA ScreenTape ladder (Agilent, catalog number: 5067-5581)

21. Saturated ammonium sulphate solution (Macklin, catalog number: A885462-100ml)

22. Chloroform (Supelco, catalog number: 02487)

**Solutions**

1. EZ-Link psoralen-PEG3-biotin stock solution (see Recipes)

2. 1% digitonin work solution (see Recipes)

3. Crosslinking solution (see Recipes)

**Recipes**

**1. EZ-Link psoralen-PEG3-biotin stock solution**

Dissolve in DMSO according to the user guide to make a 200 mM stock solution (~1.38 mg/100 μL). Store at 4 °C. Shelf-life: 4 weeks.

**2. 1% digitonin work solution**

Make a 1:5 dilution from 5% digitonin in DMSO. Store at 4 °C. Shelf-life: 1 week.

*Note: If the 5% digitonin solution precipitates out of solution, warm the solution at 95 °C for 5 min and then vortex to dissolve the precipitate. Cool to room temperature and dilute with DMSO.*

**3. Crosslinking solution**

**Table:** 

Reagent	Final concentration	Volume	Storage temperature	Shelf-life
EZ-Link psoralen-PEG3-biotin stock solution (200 mM)	2 mM	1 μL	4 °C	4 weeks
1% digitonin work solution	0.01% (81 μM)	1 μL	4 °C	1 week
Nuclease-free PBS	n/a	98 μL	RT	n/a
Total	n/a	100 μL	n/a	n/a

Quantities are per sample. Prepare fresh. To prepare 100 μL of crosslinking solution, sequentially combine 98 μL of nuclease-free PBS, 1 μL of 200 mM EZ-Link psoralen-PEG3-biotin, and 1 μL of 1% digitonin. Then, mix thoroughly by pipetting up and down at least 10 times to achieve final concentrations of 2 mM psoralen and 0.01% Digitonin.

**Laboratory supplies**

1. 10 cm Petri dish (Thermo Fisher Scientific, Thermo Scientific, catalog number: 150466)

2. 6-well plate for cell culture (CORNING, Costar, catalog number: 3516)

3. 15 mL centrifuge tube (NEST, catalog number: 601052)

4. 200 μL nuclease-free PCR tube (CORNING, Axygen, catalog number: PCR-02-C)

5. 1.5 mL nuclease-free centrifuge tube (LABSELECT, catalog number: MCT-001-150-S)

6. Biodyne B Nylon membrane, 0.45 μm, 8 cm × 12 cm (Thermo Fisher Scientific, Thermo Scientific, catalog number: 77016)

7. Filter paper

## Equipment

1. Biological safety cabinet

2. Centrifuge

3. Refrigerated centrifuge

4. UVP CL-1000L crosslinker (UVP, catalog number: 95-0228-02)

5. UVP CX-2000 crosslinker (UVP, catalog number: 95-0339-02)

*Note: These crosslinkers have different wavelengths of ultraviolet: UVP CL-1000L with 365 nm for crosslinking and UVP CX-2000 with 256 nm for crosslinking reversal.*

6. Qubit 4 fluorometer (Thermo Fisher Scientific, Invitrogen, catalog number: Q33238)

7. Oven

8. Water bath

9. Tray

10. Incubator shaker

11. X-ray camera

12. PCR thermal cycler

13. Magnetic separation device

14. Agilent 2100/4200 bioanalyzer (Agilent, catalog number: G2991BA for Agilent 4200 TapeStation)

15. Illumina NovaSeq 6000 Platform (Illumina)

## Software and datasets

1. fastqc (http://www.bioinformatics.babraham.ac.uk/projects/fastqc/, version: 0.12.1)

2. pear (https://github.com/tseemann/PEAR, version: 0.9.6)

3. fastp (https://github.com/OpenGene/fastp, version: 0.24.3)

*Note: fastqc, pear, and fastp can be installed from Anaconda (channel: bioconda). This protocol describes the core analytical functions, which are maintained in the latest stable versions of these tools. Users are encouraged to install and utilize the latest versions to ensure compatibility and access to the most recent updates.*

4. hyb package (https://github.com/gkudla/hyb)

5. COMRADES package (https://github.com/gkudla/comrades)

6. unafold-3.8 (https://www.unafold.org)

7. R 4.3.2 and R packages: data.table, dplyr, ggplot2, tidyr, reshape2

*Note: The listed R packages can be installed from CRAN using install.packages(). For packages from other sources like Bioconductor, please follow the respective official installation guidelines. We recommend using the latest stable versions for optimal compatibility.*

## Procedure

**A. Cell and virus culture**

1. Use a 10 cm Petri dish to culture cells to 70%–80% confluence. Select an appropriate MOI for infection based on the virus type (for HIV, we set a MOI of 0.15), and collect culture samples during the exponential growth phase.

2. Centrifuge at 150× *g* for 5 min to separate cells (pellet) from virus (supernatant) in the culture.

3. Transfer the viral supernatant and centrifuge at 200× *g* for 10 min at 4 °C to remove cellular debris.

4. Mix the supernatant from step A3 with an equal volume of saturated ammonium sulphate solution. Pipette up and down, then incubate at 4 °C for 1 h to obtain a precipitate of viral particles.

**B. Crosslinking and RNA extraction**

1. Resuspend both the viral particles and cell pellets from each sample in 100 μL of crosslinking solution, dispense into a 6-well plate, and label each sample.

2. Gently shake the 6-well plate and incubate at 37 °C for 5 min.

3. Place the 6-well plate on ice, transfer it to the CL-1000L crosslinker, remove the plate cover, and irradiate with 365 nm UV light for 20 min. The 6-well plate should remain on ice.

4. Purify RNA using the RNeasy Mini kit following the manufacturer’s instructions. Briefly, lyse samples in buffer RLT. Mix the lysate with 70% ethanol and apply it to the RNeasy silica spin column to bind the RNA. Treat the column with DNase I to remove genomic DNA contamination, followed by washing with buffer RW1 and buffer RPE to remove impurities. Finally, elute the purified RNA in 30 μL of RNase-free water. Use 1 μL aliquots for quality control, and store the remaining RNA at -80 °C.

**Pause point:** Purified RNA can be stored at -80 °C for 2 weeks.

**Caution:** All procedures involving live HIV-1, including cell and virus culturing, crosslinking, and RNA purification, **must** be conducted in a BSL-3 (P3) or higher certified biosafety laboratory.

**Figure 1.**
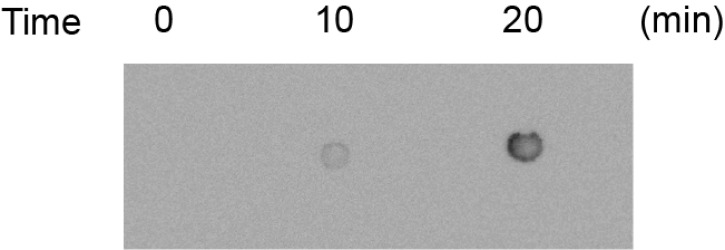
Representative result of conducting the crosslinking efficacy assessment. The image shows a chemiluminescent detection of biotinylated RNA following UV crosslinking to the nylon membrane. A good result is characterized by clear, high-intensity signals at the expected positions, indicating that the interacted RNA fragments were successfully crosslinked. The background is uniform and low, ensuring a high signal-to-noise ratio. Bad crosslinking would result in the absence of these signals.

5. Take 1 μL of quality-control RNA and quantify using Qubit. Based on RNA concentration, aliquot 200 ng of quality-control RNA per sample into a new 200 μL nuclease-free PCR tube. Resuspend in nuclease-free water to a final volume of 2 μL for use in dot blot.

6. Immerse the Biodyne B nylon membrane in 20 mL of Milli-Q water for 1 min and then transfer to 1× PBS and equilibrate for 5 min.

7. Remove the membrane from the container with Milli-Q water, gently blot the surface dry with filter paper, and allow to air-dry completely at room temperature for 5 min.

8. Spot 2 μL of the quality-control RNA (from step B5) for each sample onto the membrane. Include appropriate positive and negative controls on the same membrane.

9. Remove the membrane and allow it to air-dry horizontally at room temperature for 2 min until completely dry.

10. Sandwich the membrane between two clean filter papers and place in an oven preheated to 80 °C. Bake at 80 °C for 2 h, then allow the membrane to cool to room temperature.

11. Assess crosslinking efficacy using the chemiluminescent nucleic acid detection module, according to the manufacturer’s instructions. Briefly, incubate the membrane in blocking buffer with gentle shaking to prevent nonspecific binding. Prepare a 1:300 dilution of the stabilized streptavidin-horseradish peroxidase (HRP) conjugate in blocking buffer and incubate the membrane in this solution with gentle shaking. Wash the membrane four times with 1× wash solution to remove unbound HRP conjugate and incubate the membrane in substrate equilibration buffer. Prepare the chemiluminescent substrate working solution and incubate the membrane in the working solution. Remove excess solution and wrap the membrane in plastic wrap (avoiding bubbles). Expose to an X-ray film or a CCD imaging system to capture the signal. Use a typical successful crosslinking result as a reference (Figure 1).

**Caution:** Avoid directly exposing the reaction system to intense light while incubating the membrane in chemiluminescent substrate working solution.

**C. RNA fragmentation**

1. Calculate the quantity of RNase-free water “n” based on the volume of RNA and the final volume. Assemble the RNA fragmentation system for each sample in a 200 μL nuclease-free PCR tube (Table 1):

**Table 1.** Recipe for the RNA fragment reaction mix.

Component	Quantity
RNase III buffer (10×)	2 μL
RNA	1 μg
RNase-free water	n
10× MnCl_2_	2 μL
RNase III	2 μL
Total	20 μL

Mix gently by pipetting up and down, then centrifuge briefly to collect the solution at the bottom of the tube before incubation.

**Figure 2.**
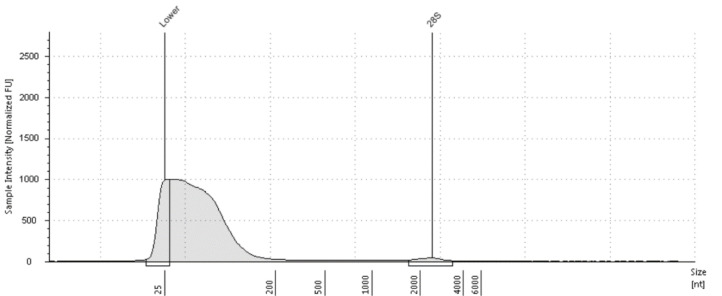
Typical example of a peak diagram in Agilent 4200 of successful fragmentation of RNA samples. A diffuse peak with a size of approximately 200 nt can be recognized.

2. Incubate at 37 °C for 5 min and then immediately proceed to the purification of fragmented RNA.

3. Add 40 μL of (2×) MagicPure RNA beads to each sample. Follow the manufacturer’s instructions.

4. Withdraw 3 μL of purified product from each sample for quality control. Divide the remainder equally into two portions, labeled *control* and *ligation* groups.

5. Quantify fragmented RNA using Qubit. Detect fragment size using Agilent Bioanalyzer. Successful fragmentation is indicated by a diffuse peak, as shown in Figure 2.

*Note: For Agilent high-sensitivity RNA ScreenTape analysis, for each analysis, a fixed volume of 2 μL was used. The RNA sample concentration could be adjusted to be within the manufacturer's optimal range of 1,000–10,000 pg/μL to ensure accurate quantification and reliable RNA integrity number equivalent (RINe) values.*

**D. Proximity ligation and crosslink reversal**

1. For each sample, calculate the quantity of RNase-free water “n” based on the volume of RNA and the final volume, and assemble the ligation system in a 1.5 mL nuclease-free centrifuge tube as follows (Table 2):

**Table 2.** Recipe for the proximity ligation reaction mix.

Component	Quantity
T4 RNA ligase buffer	20 μL
10 mM ATP	20 μL
SUPERase·In	1 μL
RiboLock RI	5 μL
T4 RNA ligase 1	20 μL
T4 PNK	5 μL
RNA	200 ng
RNase-free water	n
Total	200 μL

Mix gently by pipetting up and down. Centrifuge briefly to collect the solution at the bottom of the tube before incubation.

2. Incubate at 16 °C for 16 h.

**Pause point:** In practice, overnight incubation is permissible.

3. Upon initiation of incubation, place the control sample on ice. Open the lid and position it within the CX-2000 crosslinker. Irradiate with 254 nm ultraviolet for 5 min to reverse crosslinking.

4. Upon completion of ligation sample incubation, purify the ligated RNA using the miRNeasy Micro kit. Add 800 μL of QIAzol lysis reagent to the reaction mixture. Perform phase separation by adding 200 μL of chloroform and centrifuging at 12,000× *g* for 15 min at 4 °C. Carefully transfer the upper aqueous phase to a new tube. Subsequently, add 1.5 volumes of 100% ethanol to the sample and apply it to the RNeasy MinElute spin column. Wash the column with buffer RWT and buffer RPE, and elute the purified, ligated RNA in 14 μL of RNase-free water. Quantify the purified product using Qubit.

5. Irradiate the ligation sample with 254 nm UV light for 5 min to reverse crosslinking.

**E. Library preparation and sequencing**

1. Before library preparation, determine RNA concentration using Qubit and assess fragment distribution via Agilent Bioanalyzer.

2. Aliquot 50 ng per sample and adjust all RNA volumes to 8 μL using nuclease-free water.

3. Perform library preparation following the SMARTer Stranded Total RNA-Seq kit v2 (Pico Input Mammalian User Manual). Briefly, reverse-transcribe total RNA into first-strand cDNA using random primers, which incorporate a template-switching oligo at the 3′ end of first-strand cDNA. Add Illumina adapters and indexes during the initial PCR amplification. Purify the RNA-Seq library by AMPure beads and cleave cDNA fragments originating from mammalian ribosomal RNA (rRNA) and mitochondrial RNA using the ZapR v2 enzyme in the presence of specific R-Probes. Enrich the final library, composed of non-ribosomal cDNA, through a second round of PCR. Purify the library with AMPure beads and validate its quality using Agilent Bioanalyzer and Qubit.

4. Perform HIV fragment enrichment on cDNA libraries using the TargetSeq One Hyb & Wash kit with the T548XV1 probe set designed for the HIV genome. Follow the manufacturer's operating instructions as summarized below:

a. Thaw the Hyb human block, RNase block, and TargetSeq blocking oligo and TargetSeq target probes on ice. Thaw the TargetSeq One Hyb buffer at room temperature (dissolve any precipitate at 37 °C if necessary).

b. Mix the concentrated RNA library with the blocker mix (Hyb human block, blocking oligo, RNase block, nuclease-free water, and TargetSeq target probes in the Hyb buffer to a total volume of 30 μL).

c. Incubate the reaction in a thermal cycler using the following program: 80 °C for 5 min, followed by a hold at 50 °C for 16–24 h (heated lid at 85 °C) to allow specific hybridization of probes to the target regions.

d. Thirty minutes before hybridization is complete, equilibrate the TargetSeq Cap beads to room temperature. Remove the storage buffer and wash the beads three times with binding buffer using a magnetic rack. Resuspend the beads in binding buffer.

e. Transfer the prepared beads to the hybridization mixture (maintained at 50 °C). Incubate at room temperature for 30 min with rotation to bind the biotinylated probe-target hybrids to the streptavidin beads.

f. Separate beads using a magnetic rack and remove the supernatant. Wash molecules with wash buffer 1 at room temperature for 15 min with rotation.

g. Perform high-stringency washes using preheated TargetSeq One Wash Buffer 2 v2 at 50 °C for 10 min. Repeat this high-stringency wash step according to the manufacturer's optimization guidelines to remove nonspecific binding. Transfer the entire bead suspension to a new PCR tube. Capture the beads on a magnetic rack and discard the supernatant. Wash the beads with 200 μL of 80% ethanol for 30 s. Carefully remove all residual ethanol and air-dry the beads for 3–5 min at room temperature.

h. After the final wash, resuspend the air-dried beads in 24 μL of nuclease-free water, and add the post-PCR master mix and primers directly to the beads. Perform PCR amplification (cycle number N is determined based on the initial library input) and purify the amplified product using AMPure XP beads to obtain the final enriched library.

5. Conduct paired-end sequencing (PE 150) on the Illumina Novaseq 6000 platform. Output format: FASTQ.

**F. Chimeras and interaction calling**

1. Use fastqc to check sequencing quality.

2. For each sample’s fastq files, use PEAR to merge overlapping reads, combining all files into pseudo-SE sequencing data. Then, use fastp to filter low-quality reads and trim adapters. Use the following commands:

pear -e -j <threads> -f sample_forward.fastq.gz -r sample_reverse.fastq.gz -o Sample.PEAR

cat Sample.PEAR.assembled.fastq Sample.PEAR.unassembled.forward.fastq Sample.PEAR.unassembled.reverse.fastq > Sample.PEAR.fastq

fastp -i sample.PEAR.fastq -o sample.PEAR.fastp.fastq -h fastp.html -w <threads> -a <adapter_sequence>

3. Obtain the reference genome sequence (in FASTA format) for the virus under study (e.g., HIV-1). Use the following command to create a corresponding database:

hyb detect in= sample.PEAR.fastp.fastq db=Reference/database/dir qc=none

4. Construction of intra-molecular contact matrix: Process the chimeric reads from .hyb files to generate a normalized, merged intra-molecular contact matrix. This process utilizes a suite of custom R functions, publicly available at https://ngdc.cncb.ac.cn/biocode/tools/BT007456, to handle individual replicates, normalize them, and prepare them for visualization.

a. Generate a raw contact matrix for each biological replicate. Use the call_intra_matrix() function on each replicate’s .hyb file. This function bins the RNA sequence, maps chimeric reads to create a matrix, and outputs the raw interaction counts.

<R>

# Example for one replicate

call_intra_matrix(hybfile = "sample_hyb_file.hyb", removeOverlap = TRUE/FALSE, Bait_RNA = Bait_RNA, matrixfile = "sample_matrix.cpm")

b. Normalize and merge the matrices. Normalize each matrix by its total number of mapped reads. Then, merge the normalized matrices (e.g., from two replicates) to create a final, robust interaction matrix.

c. Convert the final matrix to a .cdt file for visualization. Use the matrix2cdt() function to format the merged matrix.

<R>

**Figure 3.**
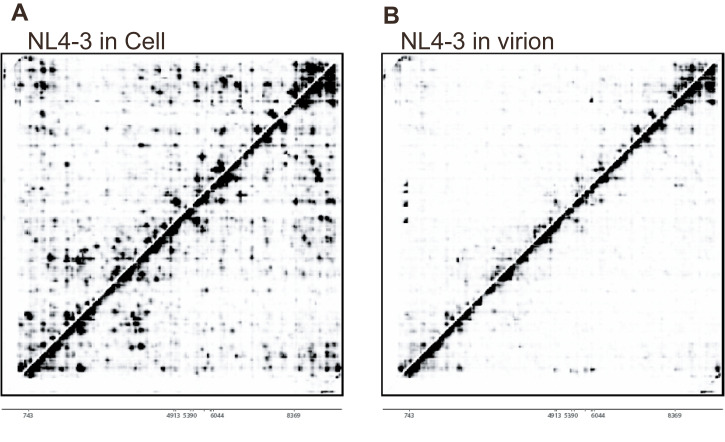
Visualization of a typical heatmap of the contact matrix. Heatmaps of the HIV-1 NL4-3 RNA contact matrix, showing interactions in vivo (A) and in virion (B). The X and Y axes represent the linear sequence coordinates of the RNA from 5′ to 3′. The color intensity indicates the normalized interaction frequency between two genomic regions. High-intensity signals represent strong interactions, indicating that these regions are spatially proximal. The heatmap shown here was generated by merging data from two normalized biological replicates and was visualized using Java TreeView software. This figure is adapted from our previous publication: Zhang et al. [1], Mapping HIV-1 RNA Structure, Homodimers, Long-Range Interactions and persistent domains by HiCapR, originally published in *eLife*, https://doi.org/10.7554/eLife.102550.3.

matrix2cdt(matrix = merged_contact_matrix, levels = levels, contrast = 1, cdtFileName = "Final_Matrix.cdt")

d. Visualize the heatmap. Open the resulting .cdt file with visualization software such as Java TreeView to generate the final heatmap, as shown in Figure 3.

5. Folding with constraints calculated from base pair score for 1000 times requires the computer cluster running qsub:

comradesMakeConstraints -i <hyb_file.hyb> -f <reference.fasta> -b <start> -e <end>

qsub comradesFold -c <hyb_file.start-end_folding_constraints.txt> -i <reference_start-end.fasta> -s 1

**Figure 4.**
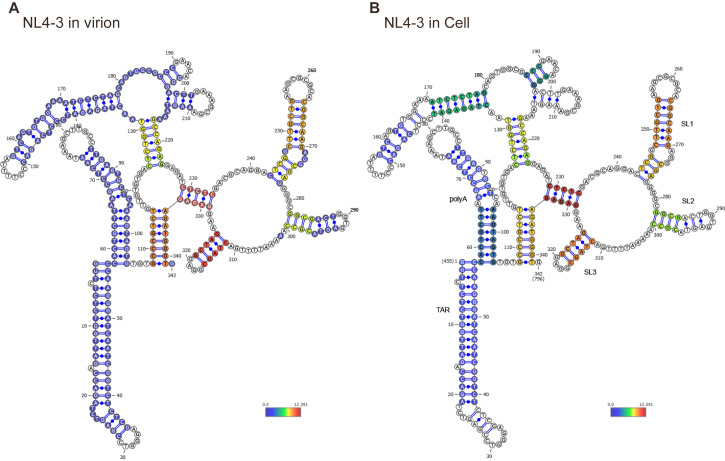
Secondary structure prediction result of the target RNA constrained by base pair scores. Representative 2G dimer secondary structures of the HIV-1 NL4-3 5’-UTR, predicted for in virion (A) and in vivo (B) conditions. We folded the target RNA using the base pair scores as constraints to generate an ensemble of potential secondary structures. Based on base pair scores and previously reported structures, the most representative structure is selected. That structure is visualized using VARNA and colored according to base pair scores. This figure is adapted from our previous publication: Zhang et al. [1], Mapping HIV-1 RNA Structure, Homodimers, Long-Range Interactions and persistent domains by HiCapR, originally published in *eLife*, https://doi.org/10.7554/eLife.102550.3.

Model the secondary structure of the target RNA using the COMRADES software pipeline. First, quantify the experimental evidence for each potential base pair by calculating the “base pair score” using the comradesMakeConstraints tool based on chimeric reads from the .hyb files. Use these scores as constraints to guide the thermodynamic energy minimization process. Subsequently, perform RNA folding using the comradesFold tool (which utilizes the UNAfold algorithm) to generate an ensemble of potential secondary structures (e.g., generate 1,000 candidate structures). From this ensemble, select the optimal structure for visualization based on two criteria: the highest total base pair score and topological consistency with well-characterized functional domains previously reported in the literature. Finally, visualize the selected structure (in .ct format) using VARNA, applying a color map to nucleotides based on their base pair scores to represent interaction confidence, as shown in Figure 4.

## Validation of protocol

This protocol has been used and validated in the following research article:

• Zhang et al. [1]. Mapping HIV-1 RNA Structure, Homodimers, Long-Range Interactions and persistent domains by HiCapR. *eLife*. 13: RP102550. https://doi.org/10.7554/eLife.102550.3

The protocol we performed here was optimized from our previous simplified SPLASH protocol and used in the following research article:

• Zhang et al. [10]. In vivo structure and dynamics of the SARS-CoV-2 RNA genome. *Nat Commun.* 12(1): 5695. https://doi.org/10.1038/s41467-021-25999-1

## General notes and troubleshooting

**Troubleshooting**

**Problem 1:** The RNA content or purity of the virus sample is too low.

Possible cause/solution: The viral load is too low; concentrate the viral supernatant and increase DNase treatment to eliminate DNA.

**Figure 5.**
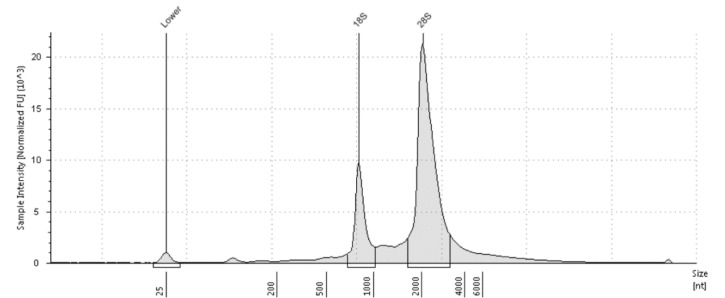
Two major peaks in the Agilent 4200 Bioanalyzer. Such a result typically indicates that the fragmentation reaction was unsuccessful.

**Problem 2:** Only a few main peaks are visible in the Agilent Bioanalyzer, as shown in Figure 5.

Possible cause: The RNase III treatment time was insufficient, or the enzyme became inactivated. Extend the digestion time or use new enzymes.

**Problem 3:** Magnetic beads are difficult to fully disperse during the purification process.

Solution: Increase the elution volume, or switch to purification using a column. You can also solve this problem by choosing non-sticky tubes.
